# Orthoflavivirus circulation in South-East Queensland, Australia, before and during the 2021–2022 incursion of Japanese encephalitis virus assessed through sero-epidemiological survey of a sentinel equine population

**DOI:** 10.1016/j.onehlt.2024.100930

**Published:** 2024-11-05

**Authors:** Nicholas K.Y. Yuen, Jessica J. Harrison, Althea S.W. Wang, Isabella E. McMahon, Gervais Habarugira, Mitchell P. Coyle, Helle Bielefeldt-Ohmann

**Affiliations:** a School of Veterinary Science, Faculty of Science, The University of Queensland, Gatton, QLD 4343, Australia; b School of Chemistry and Molecular Biosciences, Faculty of Science, The University of Queensland, Brisbane, QLD 4072, Australia; c Equine Unit, Office of the Director Gatton Campus, The University of Queensland, Gatton, QLD 4343, Australia; d Australian Infectious Diseases Research Centre, The University of Queensland, Brisbane, QLD 4072, Australia

**Keywords:** Flavivirus, Japanese encephalitis virus, Murray Valley encephalitis virus, West Nile virus, Surveillance, Outbreak, Diagnostics

## Abstract

An incursion and outbreak of Japanese encephalitis virus (JEV) was reported in Australia in 2021 and 2022, respectively. There was speculation that JEV may have been circulating in Australia unknowingly prior to the detection. In this study, we determined sero-prevalence and transmission of West Nile virus (WNV), Murray Valley encephalitis virus (MVEV) and JEV, prior to and post JEV incursion in a sentinel equine population in south-east Queensland (SEQ), Australia, using blocking ELISAs (screening test) and virus neutralisation test (confirmatory). Serum samples collected between 2018 and 2020 (prior to JEV incursion; *n* = 607) from horses residing in SEQ revealed that sero-prevalence to pathogenic orthoflaviviruses was low, specifically WNV (1.3 %; 8/607), MVEV (1.2 %; 7/607), and JEV (4.9 %; 30/607). The significantly higher prevalence of JEV (*P* < 0.05) was skewed by the high proportion of horses previously enrolled in one or more JEV vaccine studies (17/30; 56.7 %) and the unknown JEV vaccination history due to international travel (6/30; 20 %). Thirty-two foals were enrolled as sentinels to monitor for arbovirus transmissions in SEQ between 2020 and 2023. Results showed that JEV seroconversion was first detected in April 2022 (*n* = 4), with seven more seroconversions detected in the following months until November 2022. This study (i) confirms that it is highly unlikely that JEV incursion in SEQ occurred prior to February 2022; (ii) circulation of WNV in SEQ remains very low; and (iii) highlights the complexity in the interpretation of orthoflavivirus serological results. The authors propose that horses should be included as sentinels for arbovirus transmission monitoring in Australia.

## 1 Introduction

Orthoflavivirus (previously known as flavivirus), part of the family Flaviviridae, is a genus of vector-borne single plus-strand (ss)-RNA viruses. It comprises approximately 75 known viruses, organised into seven serological complexes. The Japanese encephalitis virus (JEV) serogroup complex contains three pathogenic orthoflaviviruses – Japanese encephalitis virus (JEV), Murray Valley encephalitis virus (MVEV) and West Nile virus (WNV), along with low- or non-pathogenic members such as Alfuy virus (ALFV) [1].

In Australia, MVEV was first isolated in 1951, followed by the Kunjin strain of WNV (WNV_KUN_) in north Queensland in 1960 [2]. Originally regarded as a distinct orthoflavivirus, WNV_KUN_ was re-classified as a variant of WNV after a significant degree of antigenic and genetic similarities with WNV were demonstrated [3]. JEV, which has been known to circulate in the Torres Strait Islands during 1995–2006, was first detected on the Australian mainland in the Cape York Peninsula in 1998 [4].

As mosquito-borne viruses, MVEV, WNV_KUN_ and JEV are maintained in an enzootic life cycle between waterbirds and mosquitoes [5]. In Australia, these viruses are primarily transmitted by *Culex annulirostris* (common banded mosquito) [6], and have the potential to cause encephalitis in humans and horses, both of which serve as incidental and dead-end hosts. Swine and crocodilians are amplifying hosts for JEV and WNV_KUN_, respectively [5,7,8]. In the Australian context, these orthoflaviviruses have imposed a moderate burden on human and animal health. Recent outbreaks of arboviruses, often following periods of extensive rainfall and flooding, have attracted focus on their capacity to cause significant welfare issues and economic losses.

In 1974, an outbreak of neurological disease in horses was attributed to MVEV [9]. Cases of arboviral neurological disease in horses remained sporadic until 2011 when an equine encephalitis epidemic affecting almost 1000 horses in south-eastern Australia, following a period of extensive rainfall and flooding, was attributed to mainly WNV_KUN_, with fewer cases caused by MVEV [10,11]. Prior to 2021, JEV detection had only been confirmed in far north-eastern Queensland, specifically in the Torres Strait and Cape York Peninsula [12]. After the diagnosis of JEV infection in a patient in the Northern Territory in 2021 [13], JEV was then detected in commercial piggeries causing reproductive and neurological diseases in three states in Australia in February 2022. Within three months, JEV infections were reported in 70 piggeries and 30 human cases (with five deaths) across four Australian states [14]. This outbreak was preceded by a period of extensive rainfall and associated flooding between 2021 and 2022, as Australia experienced the multi-year La Niña weather event between September 2020 and March 2023.

The south-east Queensland (SEQ) region was significantly impacted in both the 2010–2011 and 2021–2022 weather events. A previous survey, assessing the seroprevalence of orthoflaviviruses in the region's horses after the 2011 weather event, found infrequent seropositivity of about 15 % MVEV, WNV_KUN_, and ALFV, suggesting that factors other than pre-existing immunity may have contributed to the low incidence of arboviral disease in SEQ horses during the 2011 epidemic [15]. The more recent weather events in 2021–2023 and JEV outbreak prompted an update into the current seropositivity levels in SEQ and the ongoing risks to animal and human health within the region.

The present study first aimed to serve as an update on the serological status of SEQ horses with regards to pathogenic orthoflaviviruses, namely MVEV, WNV_KUN_ and JEV between 2018 and 2023, by studying racehorses residing in SEQ and a defined horse population residing in a repeatedly flood-affected area within the region, in close proximity (< 5 km) to wetlands that host a prominent waterbird population [[15], [16], [17]].

## 2 Material and methods

### 2.1 Sample collection

**Fig. 1:**
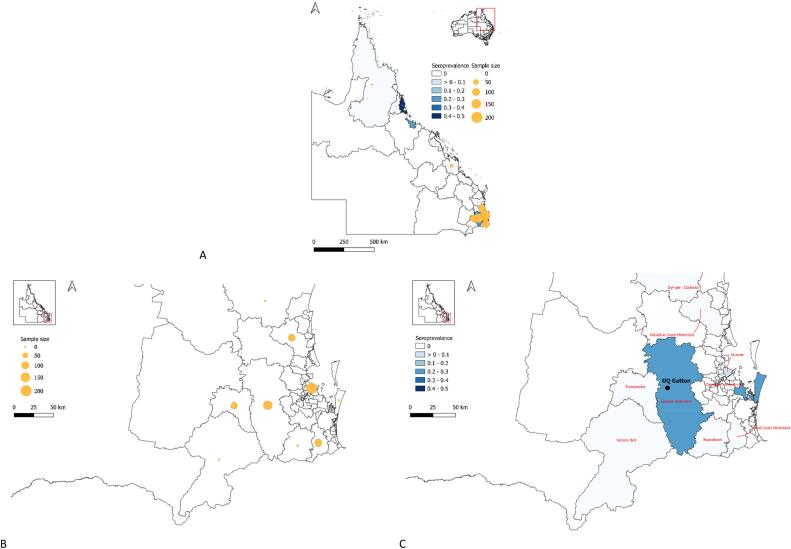
Map of QLD classified by statistical local area 3 [34] showing the distribution and apparent seroprevalence of the study population (A), the distribution of the study population in SEQ (B), and the apparent seroprevalence of the study population in SEQ (C). Black dot in Fig. 1C denotes approximate location of the UQ Gatton campus. Maps were generated using QGIS (version 3.22).

Animal ethics approval was obtained from The University of Queensland (UQ) Production Animal Ethics Committee (permit numbers SVS/344/18; 2021/AE000763) prior to commencement of the study. Over a six-year period (2018–2023), serum samples were obtained from three categories of horses: (1) racehorses attending race meetings in Queensland (QLD; 2018–2020) (*n* = 511; details described in [16]); (2) adult horses residing at UQ properties (Pinjarra Hills and Gatton; *n* = 96; 2018–2019); and (3) foals born and residing at the UQ Gatton campus, from 2020 to 2023 (*n* = 32; [17]) were enrolled. Blood samples were collected from the foals pre-suckle, post-suckle (24 h post foaling), then approximately monthly until six months of age, after which samplings were opportunistic approximately every two to three months [17]. Distribution of the study population is shown in Fig. 1. The UQ Gatton campus represents a repeatedly flood-affected area within the region, in close proximity (< 5 km) to wetlands that host a prominent waterbird population, such as egrets (*Ardeola ibis*, *Egretta* spp.), herons (*Nycticorax caledonicus*), ibises (*Threskiornis molucca*, *T. spinicollis*), magpie geese (*Anseranas semipalmata*), whistling ducks (*Dendrocygna eytoni*) and swamphens (*Porphyrio porphyrio*) [[15], [16], [17]].

Blood (5 mL) was collected from the horses via jugular venipuncture with a 21-gauge hypodermic needle into a Vacutainer® containing clot activator. The blood samples were allowed to clot at room temperature for 1 h prior to centrifugation at 1342 × *g*, at 20 °C, for 20 min. Sera were aliquoted into sterile vials and stored at −20 °C until analysis. All serum samples were heat inactivated at 56 °C for 30 min prior to laboratory testing.

### 2.2 Enzyme-linked immunosorbent assay (ELISA)

Antibodies to orthoflaviviruses in serum samples were first screened using a modified approach of a previously described epitope-blocking ELISA [15,18,19]. Briefly, U-bottomed 96-well polyvinyl chloride plates (Costar, Corning Incorporated, USA) were coated with orthoflavivirus antigen (either WNV_KUN_, MVEV or Binjari-JE chimeric virus tissue culture lysate) diluted 1:1000 in coating buffer (50 mM NaHCO3, 50 mM Na2CO3, pH 9.6) at 50 μL per well overnight at 4 °C. The plates were washed four times in wash buffer (PBS with 0.05 % Tween-20). Non-specific sites were blocked with 100 μL of blocking buffer (0.05 M Tris, 1 mM EDTA, 0.15 M NaCl, 0.05 % (*v*/v) Tween-20, 0.2 % (*w*/*v*) high nitrogen casein, pH 8.0) for 1 h at room temperature. Blocking buffer was then discarded without washing prior to addition of serum sample. Each serum sample was diluted 1:5 in blocking buffer, tested in duplicates, and incubated at 28 °C for 1 h. Known positive and negative control serum samples were added to each plate in duplicates. The plates were washed four times in wash buffer. Pan-orthoflavivirus specific monoclonal antibody (mAb), 6B6C-1 [18], was diluted 1:100 in blocking buffer and added at 50 μL per well, incubated at 28 °C for 1 h. The plates were washed four times with wash buffer. Secondary antibody, a horseradish peroxidase-conjugated goat anti-mouse immunoglobulin, pre-adsorbed against equine immunoglobulins and fetal bovine serum (Agilent DAKO, Glostrup, Denmark), was diluted in blocking buffer at 1:2000 and added in 50 μL per well to bind to the mAb at 28 °C for 1 h. The plates were washed six times with wash buffer. Bound secondary antibody was detected with 50 μL of substrate solution (by mixing 1 mM 2,2’azino-bis(3-ethylbenzthiazoline-6-sulfonic acid) (Sigma-Aldrich, Merck, Germany) and 3 mM hydrogen peroxide to citrate acid/Na2HPO4 buffer, pH 4.2), incubated in the dark, at room temperature for 1 h. Quantitative results were determined by measuring the optical density (OD) at 405 nm. OD of each duplicate was averaged. Percent inhibition was calculated:

$$\left[100-\frac{\left(\mathrm{OD}\;\text{test serum}-\mathrm{OD}\;\text{background}\right)}{(\mathrm{OD}\;\text{negative control serum}-\mathrm{OD}\;\text{background}}\right]\times 100$$

A cut-off of >30 % inhibition was determined as positive; and such samples were subsequently subjected to WNV_KUN_, MVEV and JEV-specific blocking ELISA, using mAbs 3.1112G [18], 10C6 [20] and 989 [21], respectively.

### 2.3 Virus micro-neutralisation test (VNT)

Samples positive in the screening ELISA assay were then subjected to virus neutralisation test (VNT), as described [15], for confirmatory testing and neutralising antibody titre determination. Virus strains used for VNT testing were: WNV_KUN_ (NSW 2011), MVEV (strain 1–51), and JEV (O-0883/NSW/22). Prior to the isolation of JEV O-0883/NSW/22, a chimeric virus made on an insect-specific flavivirus backbone that expresses the structural proteins of JEV (BinJ/JEV_NSW/22_-prME) [22] was used in a pseudo-neutralisation assay. VNTs were performed using Vero cells and JEV O-0883/NSW/22 as previously described [22] and an incubation period of 3 days and 115 infectious particles, as determined by back-titration. The pseudo-neutralisation assay was performed similarly to the VNT [23], with the following changes: C6/36 cells were pre-seeded 24 h prior to performing the assay at a density of 3 × 10^5^/well, and the incubation period was 5 days. After incubation, culture plates were fixed in 20 % acetone with 0.02 % bovine serum albumin in PBS. Neutralising antibody titres were determined in a fixed-cell ELISA using 4G4 monoclonal antibody [14].

### 2.4 Meteorological data

Half hourly weather data collected from the UQ Gatton Campus weather station (station no. 040082, Bureau of Meteorology, Australian Government) between 1 January 2022, and 30 June 2023, were made available for analysis. Data was summarised and visualised as described previously using Stata BE 17.0 [17].

## 3 Results & discussion

Despite the extensive flooding in SEQ in late 2010 and early 2011, the same-year epidemic of equine encephalitis in multiple states in Australia caused by a novel WNV_KUN_ strain [15,24] did not manifest in Queensland. At the time it was thought that this might be due to pre-existing immunity in the equine population, but that theory was not supported by a subsequent serological survey [15].

When JEV appeared in piggeries in SEQ, there was an anticipation that this might spill over into the equine population as well. Therefore, we conducted a serological assessment of horses sampled for another arbovirus infection, Ross River virus (RRV), during 2018–2020, i.e., prior to the JEV incursion, comprising 607 horses across two horse populations: racehorses competing and mostly residing in SEQ and horses residing on the UQ Gatton campus [16]. Only a very small subset of racehorses (1 %) had antibodies to either JEV, MVEV or WNV_KUN_ (Table 1, Table 2; Fig. 1). It should be noted that information about JEV vaccination status was not available for this cohort, but it is possible that the six horses positive in the JEV VNT had received a vaccine before attending international race-meets in Singapore, Hong Kong or Japan.

**Table 1:** Results of orthoflaviviruses antibodies detection in SEQ horses between 2018 and 2020, i.e. prior to JEV incursion.

		QRIC		UQ – unvaccinated 1		UQ – vaccinated 2	
Antigen	Assay	Positive	Negative	Positive	Negative	Positive	Negative
Pan-flavivirus	ELISA	2.5 % (13/511)	97.5 % (498/511)	48.8 % (39/80)	51.3 % (41/80)	100 % (16/16)	0 % (0/16)
WNV_KUN_	ELISA	30.8 % (4/13)	69.2 % (9/13)	20.5 % (8/39)	79.5 % (31/39)	37.5 % (6/16)	62.5 % (10/16)
	VNT	INS	INS	37.5 % (3/8)	62.5 % (5/8)	16.7 % (1/6)	83.3 % (5/6)
MVEV	ELISA	23.1 % (3/13)	76.9 % (10/13)	17.9 % (7/39)	82.1 % (32/39)	31.3 % (5/16)	68.8 % (11/16)
	VNT	66.7 % (2/3)	33.3 % (1/3)	28.6 % (2/7)	71.4 % (5/7)	60.0 % (3/5)	40.0 % (2/5)
BinJ-JEV	ELISA	53.8 % (7/13)	46.2 % (6/13)	43.6 % (17/39)	56.4 % (22/39)	100 % (16/16)	0 % (0/16)
	VNT	85.7 % (6/7)	14.3 % (1/7)	70.6 % (12/17)	29.4 % (5/17)	75.0 % (12/16)	25.0 % (4/16)

INS = insufficient samples. WNV_KUN_ = West Nile virus (Kunjin strain). MVEV = Murray Valley encephalitis virus. BinJ-JEV = Binjari-Japanese encephalitis chimeric virus.

1 UQ horses that has not been enrolled in a JEV vaccine trial prior to 2018–2020 sample collection.

2 UQ horses previously enrolled in a JEV vaccine trial in 2008 [26] or 2012 [25].

**Table 2:** Apparent prevalence of orthoflaviviruses in SEQ between 2018 and 2020, i.e. prior to JEV incursion.

	QRIC	UQ – unvaccinated 1	UQ – vaccinated 2	*P*-value⁎
WNV_KUN_	0.8 % (4/511)	3.8 % (3/80)	6.3 % (1/16)	0.0208
MVEV	0.4 % (2/511)	2.5 % (2/80)	18.8 % (3/16)	0.0001
JEV⁎⁎	1.2 % (6/511)	15 % (12/80)	75 % (12/16)	0.0001

WNV_KUN_ = West Nile virus (Kunjin strain). MVEV = Murray Valley encephalitis virus. JEV = Japanese encephalitis virus.

⁎ Kruskal-wallis test.

⁎⁎ Tested using BinJ-JEV.

1 UQ horses that has not been enrolled in a JEV vaccine trial prior to 2018–2020 sample collection.

2 UQ horses previously enrolled in a JEV vaccine trial in 2008 [26] or 2012 [25].

A significantly higher proportion of horses residing at the UQ Gatton Campus had neutralising antibodies to WNV_KUN_ (4.2 %), MVEV (5.2 %), and JEV (25 %) (Table 1, Table 2). This may in part be explained by a much older cohort of horses and hence longer arbovirus exposure [16]. Previous studies showed that not only viruses in the JEV-serogroup circulate in that area, but other orthoflaviviruses are also highly prevalent, which may explain the high antibody detection in the pan-orthoflavivirus blocking ELISA (Table 1) [15]. Moreover, this cohort included horses that had previously been enrolled in JEV vaccine studies [25,26].

Out of the 70 adult horses tested in the 2011–12 survey [15], 22 remained at the UQ Gatton Campus during sampling in 2018–19. Of those, six horses had been enrolled in a JEV-vaccine study in 2008 [26] and another eight had been enrolled in a 2012 JEV-vaccine study [25], in both instances receiving two doses of an inactivated JEV-vaccine with the adjuvant Advax. Three of the 2008-vaccinees had JEV-specific neutralising antibodies when bled in 2018–19 as did four of the 2012-vaccinees (Table 3). In addition, three adult horses bled in 2018–19 were born to mares enrolled in the 2012-vaccine study and had subsequently received two doses of the inactivated JEV vaccine when 4–5 months old and a single booster dose 10–11 months later [25]. Two of these three animals had JEV-neutralising antibodies in 2018–19 (Table 3).

**Table 3:** Neutralising antibody titres to JEV and MVEV in horses enrolled in JEV-vaccine studies 6–10 years prior.

			2012		2018–2019	
Horse	Age in 2012	JEV-vaccination, year	MVEV VNT titre⁎	JEV VNT titre⁎	MVEV VNT titre	JEV VNT titre
1	16	2008	<20	ND¶	ND¶	ND¶
2	16	2008	20	320	ND¶	160
3	11	2008	<20	40	ND¶	<20
4	16	2008	40	320	20	320
5	15	2008	<20	20	ND¶	80
6	14	Control in 2011 trial	ND¶	ND¶	ND¶	ND¶
7	5	Control in 2011 trial	<20	<20	<20	<20
8	14	2011	20	160	ND¶	480
9	11	2011	80	640	40	640
10	10	2011	20	<20	ND¶	<20
11	10	2011	<20	40	<20	20
12	9	2011	<20	<20	ND¶	<20
13	11	2011	20	20	<20	480
14	11	Control in 2011 trial	ND¶	ND¶	ND¶	80
15	13	Control in 2011 trial	<20	ND	ND¶	<20
16	13	Control in 2011 trial	ND¶	ND¶	ND¶	ND¶
17	11	Control in 2011 trial	<20	<20	ND¶	ND¶
18	4	Control in 2011 trial	ND¶	ND¶	<20	ND¶
19	12	2008	<20	20	ND¶	ND¶
20	1	2011	ND¶	ND¶	ND¶	240
21	1	2011	ND¶	ND¶	ND¶	<20
22	0.5	2012 & 2013	ND¶	20Δ	20	160

MVEV = Murray Valley encephalitis virus. JEV = Japanese encephalitis virus.

⁎ Data extracted from and previously published in [15,25,26].

¶ negative in blocking-ELISA, hence VNT not performed.

Δ remnant maternal antibodies post-suckle after birth.

There are conflicting results regarding the longevity of immunity following vaccination with inactivated virus vaccines. In humans, JEV-specific antibodies elicited by an inactivated Vero cell grown vaccine appears to last between two and six years [27,28], while in horses antibodies may wane within one year of vaccination, but immune memory persists for longer [25]. Hence, since there is no evidence of JEV transmission prior to 2022 ([12] and results below), it is highly likely that these nine animals had been recently or repeatedly exposed to one or more orthoflaviviruses known to circulate intermittently in the region, such as MVEV, WNV_KUN_ or ALFV, or orthoflaviviruses of other sero-complexes, such as Kokobera and Stratford virus [15]. This would have stimulated a recall response due to long-term T and B cell immune memory for cross-reactive epitopes [29,30].

**Fig. 2:**
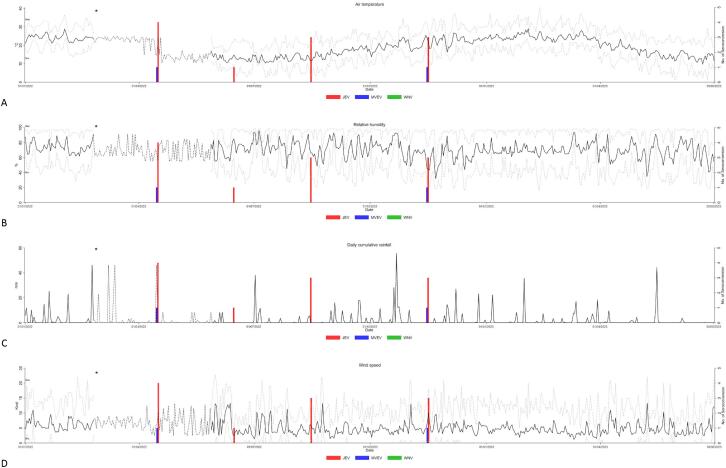
Temporal changes in the number of seroconversions and averaged daily air temperature (A), relative humidity (B), daily cumulative rainfall (C), wind speed (D) post-JEV incursion from January 2022 to July 2023 (black solid line). Data was collected at the University of Queensland Gatton Campus weather station (station no. 040082). The dotted line in black refers to imputed data (see section 2.4 and [17]). The dotted line in grey refers to averaged AM (0500–0800) or PM (1600–1900) temperature. Bar graphs represent the number of horses seroconverted to orthoflaviviruses at the various sampling time points. Asterisk (*) denotes the first detection of JEV in a piggery in Queensland.

We have previously shown high levels of RRV-specific maternal antibodies amongst foals at the UQ Gatton Campus [17]. Following waning of these antibodies below a protective level of 1:20–1:40, the foals/yearlings would get exposed to RRV within a time period of 12–15 months and all seroconversion events occurred between December and April [17]. We subjected the same set of serum samples to orthoflavivirus serology. All animals (*n* = 22) born in the Spring of 2020 or 2021 were seronegative for orthoflavivirus antibodies in the pan-orthoflavivirus blocking-ELISA between the time of birth or waning of maternal antibodies and the end of 2021. In early April 2022, four yearlings had sero-converted to JEV and by the May–November 2022 time interval seven out of 18 animals tested had antibodies to JEV, with two of these also having antibodies to MVEV – likely due to cross-reactivity (Table 4). To provide ecological context, these seroconversion events have been depicted in relation to various meteorological data, specifically air temperature, relative humidity, rainfall, and wind speed (Fig. 2). While there is no discernible weather-related risk factor, future study with larger sample size is warranted to identify risk factors associated with JEV seroconversion. None of the animals had antibodies to WNV_KUN_ as determined by the blocking ELISA, corroborating earlier findings that WNV_KUN_ circulation in SEQ is very rare [15]. It should be noted that, in this study, clinical disease was not observed in any of the animals seroconverting to orthoflavivirus.

**Table 4:** Neutralising antibody titres to MVEV and JEV in the UQ foal cohort.

Foal	Year born	Seroconversion first detected	MVEV VNT titre	JEV VNT titre
1	2020	April 2022	20	20
2	2020	August 2022	0	160
3	2020	August 2022	0	80
4	2020	August 2022	0	40
5	2020	November 2022	0	80
6	2021	April 2022	0	20–80⁎
7	2021	April 2022	20	20–80⁎
8	2021	April 2022	0	40
9	2021	June 2022	0	20
10	2021	November 2022	0	40
11	2021	November 2022	0	160

⁎ A rising titre was detected in subsequent serum samples.

Foals born in the Australian spring of 2022 (*n* = 10) had not sero-converted to JEV, MVEV or WNV_KUN_ following waning of maternal antibodies by the time of the last sampling in June 2023, suggesting orthoflavivirus circulation was minimal or non-existing in this region in the Australian fall of 2023. This agrees with the findings for RRV circulation in the very same location and animal population, of which only a couple of foals had sero-converted to RRV at the end of June 2023 despite waning of maternal RRV-specific antibodies within 4–4.5 months post colostrum uptake [17]. This suggest that there might have been less mosquito activity and arbovirus circulation in the first half year of 2023 compared to the same period in the previous two years.

The current orthoflavivirus diagnostic capabilities for veterinary species in Australia remain limited. While orthoflavivirus blocking ELISAs are routinely performed in veterinary state diagnostic laboratories, VNT is required to confirm positive results obtained from blocking ELISAs and to provide a definitive neutralising antibody titre. As a screening test, blocking ELISAs are inherently less specific than VNT and are more prone to non-specific reactivity in the sample (i.e. false positive) or cross-reactivity from other related viruses (as discussed earlier). In cases where neutralising antibodies have been detected for more than one orthoflavivirus belonging to the same sero-complex group (e.g. MVEV and JEV) without a four-fold difference in titre, the possibility of cross-reactivity from other low−/non-pathogenic orthoflaviviruses (e.g. ALFV) in the same sero-complex should be considered [14], as should co-infection [31].

## 4 Limitations

It should be noted that a few limitations present in this study. Firstly, the study population was mainly comprised of horses residing in SEQ with an uneven distribution. Therefore, results may not be representative of the entire state of QLD and is likely biased towards specific regions of SEQ. Secondly, the longitudinal study of orthoflaviviruses seroprevalence at the UQ Gatton campus has a small sample size which may limit the generalisability of the findings. However, studies of sentinel animals are very limited, and the results presented in this study are therefore of significance to public health. Lastly, as discussed earlier, co-infection and cross-reactivity of neutralising antibodies to other low- or non-pathogenic orthoflaviviruses belonging to the same serogroup should be considered as shown by an earlier sero-survey demonstrating that ALFV is prevalent at the UQ Gatton campus [15].

## 5 Conclusion

Results from this surveillance effort suggest that it is highly unlikely that transmission of JEV in SEQ occurred prior to the initial incursion event reported in February 2022. Given that the first seroconversion to JEV in horses was detected in early April 2022, this would suggest that JEV transmission in the region started sometime in March 2022. This study highlights the complexity of orthoflavivirus serological results interpretation, and care must be taken to identify potential cross-reactivity to other orthoflaviviruses in the JEV sero-complex group prior to definitive diagnosis. Furthermore, the use of horses as sentinels for the monitoring of arbovirus transmission, in particular alphavirus [17,32] and orthoflavivirus [15,33], should be considered.

## Funding statement

Funding was received from the Australian Infectious Diseases Research Centre (to H.B.O., 2018–20), the Peter and Mary Ellen Stone Memorial Fund (to H.B.O., 2018–19), and a donation from BIOHMPATHOLOGY. N.K.Y.Y. and G.H. were recipients of UQ Research Training Scholarships during most of this project. J.J.H. is supported by an ARC-DECRA award (DE230101284).

## CRediT authorship contribution statement

**Nicholas K.Y. Yuen:** Writing – review & editing, Writing – original draft, Methodology, Investigation, Formal analysis, Data curation, Conceptualization. **Jessica J. Harrison:** Writing – review & editing, Methodology, Data curation. **Althea S.W. Wang:** Writing – review & editing, Data curation. **Isabella E. McMahon:** Writing – review & editing, Methodology, Data curation. **Gervais Habarugira:** Writing – review & editing, Methodology. **Mitchell P. Coyle:** Writing – review & editing, Investigation. **Helle Bielefeldt-Ohmann:** Writing – review & editing, Writing – original draft, Supervision, Project administration, Methodology, Investigation, Funding acquisition, Conceptualization.

## Declaration of competing interest

H.B.O. is the proprietor of the consultancy firm BIOHMPATHOLOGY. Data included in this publication are based on a patent (WO/2018/176075), on which H.B.O and J.J.H are inventors. All other authors declare no conflict of interest.

## Data Availability

All data have been presented in the manuscript.
